# Violent typologies among women inpatients with severe mental illness

**DOI:** 10.1007/s00127-016-1280-x

**Published:** 2016-09-03

**Authors:** Richelle Schaefer, Matthew Broadbent, Matt Bruce

**Affiliations:** 1 King’s College London, Strand, London, WC2R 2LS UK; 2 J 5, 68159 Mannheim, Germany

**Keywords:** Severe mental illness (SMI), Disruptive behaviours, Physical violence, Deliberate self-harm (DSH)

## Abstract

**Purpose:**

Extant severe mental illness (SMI) and physical violence literature focus disproportionately on community-based men samples. To address this empirical imbalance, the current study explored violence towards others and oneself among women inpatients with SMI. As those with SMI are more likely to be victims than perpetrators of violence, victimisation was also an important factor assessed in this study.

**Methods:**

The study used a quantitative within-subject cross-sectional design. Data were extracted from 5675 inpatient women cases between 2009 and 2013.

**Results:**

Women with a manic disorder (without psychotic features) were 4.5 times, whilst those with psychotic disorders were 2 times, more likely to be physically violent to others compared to those with major mood disorders. Conversely, women with a major mood disorders were 4.8 times and 7.5 times more likely to engage in violence towards oneself (deliberate self-harm), compared to those with psychotic disorder and manic disorders, respectively. The past victimisation increased the likelihood of later physical violence.

**Conclusion:**

The data illuminate differential risk factors among women inpatients with SMI that may help predict violence occurring towards others and oneself and allow gender comparisons with the established literature.

## Introduction

Violent behaviours perpetrated by inpatients with a severe mental illness (SMI) are a significant problem in psychiatric facilities [1–3] and to a lesser (debatable) extent, within the community [4–6]. In broad terms, SMI can be subdivided into two long-term psychiatric disorders: psychotic (e.g., schizophrenia, schizoaffective, substance-induced psychosis) and non-psychotic major mood (e.g., depression, bipolar and anxiety) disorders [7, 8]. Few studies have focused on the relationship between violence and SMI exclusively among women inpatients. This is surprising given that the available evidence suggests that women with an SMI have a higher likelihood of physical violence perpetration compared to their men counterparts [9–12]. According to Dinaker and Sobel [13], women accounted for over half of the physical violent acts within an inpatient facility despite accounting for only one quarter of the total sample. The presence of a manic episode also appears to be a primary determining factor for physical violence, as shown by Binder and McNiels [14] hospital record study of 253 inpatients. These results complemented a later study, which showed out of 260 physically violent inpatients (of 1025 inpatients), the most powerful unique predictor of physical violence was either bipolar disorder (type unspecified) or personality disorder (PD) in acute stay inpatients (gender failed to predict physical violence) [15]. A further study showed that those with bipolar (with current manic episode) disorder had a five-fold increase of self-reported violence and/or court convictions for violence offending, similar to those with psychotic disorders [16], complementing Fazel et al.’s meta-analytic findings [17].

Among individuals with SMI, aggression towards oneself (e.g., deliberate self-harm) is often overlooked in the literature pertaining to inpatient violence. The current research suggests that deliberate self-harm (DSH) is more strongly related to major mood disorders than psychotic disorders [18, 19], with limited evidence suggesting that positive psychotic symptomatology may also influence self-harm and suicidal behaviours [20].

Disruptive, destructive, and violent behaviours exhibited by psychiatric inpatients are rarely directly attributable to SMI in isolation. Accordingly, additional explanatory factors have been found to moderate this relationship including: substance misuse [12, 21]; personality disorder, specifically the presence of borderline (BPD) and antisocial personality disorder (ASPD) [22]; ethnicity, as racial inequalities are well documented within the health system in Britain [23, 24], and as such, ethnicity is an important (and politically contentious) variable that is often included but rarely explored within research studies [25]; and social vulnerabilities (e.g., victimisation and homelessness), as these factors increase ones risk for physical violence [26–28], particularly in those with psychotic disorders [29].

The present study hypothesised that: (1.1) those with psychotic and manic disorders will engage in more disruptive and violent behaviours compared to those with all major mood disorders; (1.2) the relationship between these disorders and disruptive and violent behaviours will be moderated by substance misuse, ASPD, ethnicity, and victimisation; (2.1) those with major mood disorders will engage in more deliberate self-harm compared to those with psychotic and bipolar (with current manic episode) diagnoses; and (2.2) the relationship between major mood disorders and deliberate self-harm will be moderated by substance misuse, BPD, ethnicity, and victimisation.

## Methods

### Participants and data extraction

The data currency in this study was not patient level, but rather admissions level. Therefore, every new admission was coded as a new case, regardless if the patient was admitted previously. In this study, there were 5675 women cases—53 % participants were admitted once, 20.7 % were admitted twice, 10.8 % were admitted three times, 6.1 % were admitted four times, and 9.4 % inpatients were admitted 5–16 times. All participants were active South London and Maudsley (SLaM) National Health Service (NHS) Foundation Trust inpatients between January 1, 2008 to December 31, 2013. To be selected for this cohort, each inpatient must have had a primary SMI diagnosis (according to the *ICD*-*10*) and must have been an adult at the first referral date (over 18 years of age). The information within each inpatient’s full electronic clinical record is routinely updated by SLaM employees. These files are accessible via the BRC Clinical Records Interactive Search (CRIS) database, which is supported by the NIHR Biomedical Research Centre for Mental Health BRC Nucleus at the SLaM NHS Foundation Trust and Institute of Psychiatry, King’s College London jointly funded by the Guy’s and St Thomas’ Trustees and the SLaM Trustees. All the measurements included for each inpatient case were within a 2-week to 3-month period of that inpatient’s referral date. The SMI diagnosis must have been documented from the admission date up until the next admission.

This study used a within-subject cross-sectional design, with all measurements documented within a 3-month time frame of the inpatients admission date. Therefore, temporal proximity between SMI (i.e., psychotic symptoms and severe symptoms) and outcome variables (i.e., disruptive behaviours, physical violence, and deliberate self-harm) was accounted for in the design and statistical analyses of the current study. This will help to add to clarity this field of research, as there are inconsistent results when addressing psychotic disorders and physical violence stem from statistical designs not accounting for causality or temporal proximity [30], as active psychotic symptoms have been proposed to drive the relationship between psychotic illnesses and subsequent violent acts [31, 32]. Given this study design, there is also a minimal chance for selection bias [33].

### Measures

#### Severe mental illness

Primary SMI diagnoses were based on the ICD-10 [34] and were extracted from medical records using the CRIS search tool. All eligible cases (*N* = 5675) were included who met the *ICD*-*10* diagnosis for: major mood disorders (without psychotic features) (*n* = 1635); psychotic disorders (*n* = 3594); and (2) manic disorders (without psychotic features) (*n* = 446) [coding: (0) major mood disorders: F20.4, F31, F31.3, and F31.6-F39); (1) psychotic disorders: F20–29, F30.2, F31.2, F31.5, F32.3, and F33.3; and (2) manic disorders (i.e., F30, F30.0, F30.1, F30.9, F31.0, and F31.1 (refer to ICD-10 for specific names)].

#### Demographics

The basic information was collected for each inpatient episode, including age, marital status (i.e., single, married/cohabiting, and divorced/widowed), homelessness (yes/no), and ethnicity (white—British, Irish, any other White background; black—African, Caribbean, any other Black background; Asian—Indian, Bangladeshi, Pakistani, Chinese, any other Asian background; Biracial—White and Black African, White and Asian, any other biracial background).

#### Substance misuse

A measure of substance misuse was extracted from the Health of the Nation Outcome Scales (HoNOS) [35, 36].

#### Personality disorder

The presence of borderline personality disorder (BPD) and antisocial personality disorder (ASPD) was extracted from in-text case notes. Both variables were coded separately as binary (0 and 1) variables. If there was no mention of BPD and/or ASPD, the case was coded as negative (i.e., not displaying characteristics of BPD or ASPD).

#### Victimisation

Coding was based on qualitative notes within the CRIS database, using a programme titled ‘TextHunter’ developed by Mr. Richard Jackson. Within this programme, victimisation was extracted using the code word: [Vv]ictim. Victim was not coded if notes documented: inpatient had delusions of being victimised, inpatient “plays of victim/feels victimised”, and inpatient said she was a victim of something abstract (i.e., “victim of the system/victim of substance misuse”. Victimisation was coded as present if notes documented: inpatient made a personal statement about being victimised (not due to delusions), inpatient needed/attended a victim support service, there was a recorded history of domestic violence or stalking, and/or historical victimisation was noted; precision: 0.678; recall: 0.871). Positive victimisation was coded to include all forms of victimisation, as defined in Teplin, McCelland, Abram, and Weiner’s [37] study and did not differentiate between violent and non-violent victimisation, congruent with later findings [38]. This variable was coded using TextHunter, as it was not available within documented questionnaires within the CRIS database.

#### Distruptive behaviours (HoNOS) [35]

Item 1 of 12 was used ‘Overactive, aggressive, disruptive behaviour’ that is scored from 0 (no problem) to 4 (severe to very severe problem). Scores of 3 and 4 were subsequently coded as positive for disruptive behaviours.

#### Violent behaviours (TextHunter)

The in-text variable of violent behaviour towards others was extracted from the CRIS database using TextHunter. Data were extracted using the terminology used within the History of Aggressive Behaviour Form (HABS; developed in the MacArther Risk Assessment Study questionnaire) [39]. Based on the key words, each case was coded as either (0) no recent violent behaviour or (1) recent serious violent behaviour.

#### Deliberate self-harm (HoNOS) [35]

As with the physical violence variable, a measure of self-harm was also extracted (item 2 of 12) ‘non-accidental self-injury’. Each item is scored from 0 (no problem) to 4 (severe to very severe problem). Scores of 3 and 4 were subsequently coded as positive for deliberate self-harm behaviours.

### Statistical analysis

To assess the relationships between variables specified in this study’s hypotheses, univariate analyses and multivariate analyses were conducted. First, univariate analyses were performed to compare demographic, clinical factors (i.e., SMI, substance misuse, PD), and outcome variables (disruptive, violent, and deliberate self-harm behaviours) across major mood, psychotic, and manic disorder groups, using Chi-square analyses. Thereafter, binary logistic regression models were calculated to identify the degree to which the three categories of SMI predicted the three dependent variables and the extent to which confounding variables modified these predictions at the *p* value of 0.01 or less (the alpha level of 0.01 was used to decrease the type 1 error). Based on theory and previous findings, covariates were entered into multivariate analyses in five blocks: (1) SMI; (2) SMI plus control variables; (3) SMI plus control variables plus substance misuse; (4) SMI plus control variables plus substance misuse plus PD; (5) SMI plus control variables plus substance misuse plus PD plus ethnicity; and (6) SMI plus control variables plus substance misuse plus PD plus ethnicity plus victimisation. For brevity, the final step (6) will be shown. Statistical analyses were performed using the IBM SPSS Statistics version 22.0 for Windows software (SPSS Inc. Chicago, IL, USA).

## Results

Table 1 illustrates demographic and clinical characteristics across SMI groups. Based on the *ICD*-*10* criteria, 1635 inpatients (28.8 %) had a major mood disorder (without psychotic features), 3594 (63.3 %) had a psychotic disorder, and 446 (7.9 %) had a manic disorder (without psychotic features). Regarding age, there was a significant (although clinically irrelevant) difference between major mood and psychotic groups. Those with psychotic disorders were more likely to be single (63.7 %), whereas those with manic disorders were most likely to be married/cohabiting (22.2 %), not far greater those with major mood disorders (21.9 %). Those in the major mood disorder group had the highest percentages of divorcees/widowed (25.9 %) individuals. The percentage of those who were homeless at admission was low across all groups, with an overall percentage of 2.2 %, although there was still a significant different present, with the psychotic group having the highest percentage (1.9 %).

**Table 1:** Demographic, clinical, and outcome variables of total sample and SMI disorder groups

	Total sample (*n* = 5675)	Major mood (*n* = 1635)	Psychosis (*n* = 3594)	Mania (*n* = 446)	*F/X* ^2^	*p*
	*N *(%)	*N* (%)	*N* (%)	*N* (%)		
Age [M (SD)]	44 (15.8)	46 (17.0)	44 (15.3)	44.5 (15.0)	12.02	<0.001
Martial Status					83.04	<0.001
Single	3393 (59.8)	836 (51.1)	2289 (63.7)	268 (60.1)		
Married	1030 (18.1)	358 (21.9)	573 (15.9)	99 (22.2)		
Divorced	1196 (21.1)	423 (25.9)	699 (19.4)	74 (16.6)		
Not Disclosed	56 (1)	18 (1.1)	33 (0.9)	5 (1.1)		
Ethnicity					608.94	<0.001
White	2480 (43.7)	1116 (68.3)	1170 (32.6)	194 (43.5)		
Black	2463 (43.4)	359 (22.0)	1924 (53.3)	180 (40.4)		
Asian	329 (5.8)	65 (4.0)	226 (6.3)	38 (8.5)		
Biracial	403 (7.1)	95 (5.8)	274 (7.6)	34 (7.6)		
Homelessness	127 (2.20)	11 (0.7)	106 (1.9)	10 (0.2)	26.62	<0.001
Substance misuse	586 (10.3)	212 (13.0)	326 (9.1)	48 (10.8)	18.52	<0.001
Borderline PD	651 (11.5)	327 (20.0)	276 (7.7)	48 (10.8)	168.21	<0.001
Antisocial PD	34 (0.6)	3 (0.2)	30 (0.8)	1 (0.2)	9.15	0.010
Victimisation	491 (9.3)	98 (5.9)	343 (9.5)	40 (9.0)	18.41	<0.001
Disruptive behaviour	1385 (24.4)	240 (14.7)	952 (26.5)	193 (43.3)	178.36	<0.001
Violent behaviour	523 (9.2)	73 (4.5)	371 (10.3)	79 (17.7)	87.86	<0.001
Deliberate self harm	764 (13.5)	502 (30.7)	240 (6.7)	44 (4.9)	586.61	<0.001

*M* mean, SD standard deviation, *PD* personality disorder

With respect to the clinical (covariate) variables, those in the major mood group had the highest percentage of substance misuse (13 %), followed by the mania group (10.8 %), and finally by the psychotic group (9.1 %). Those in the major mood group also had the highest percentage of BPD (20 %). The percentage of ASPD was extremely low across all groups, with the psychotic group having slightly higher percentage (0.8 %), compared to 0.2 % in the other two groups. With regard to ethnicity, those in the major mood group had the highest percentage of White inpatients (68.3 %), whereas those in the psychotic group had the highest percentage of Black inpatients (53.3 %).

With respect to all three outcomes variable, there were significant differences between groups. Those in the mania group had the highest percentage of disruptive behaviour (43.3 %) and violent acts (17.7 %). Those in the major mood group had the highest percentage of deliberate self-harm (30.7 %) compared to the other two groups.

### Disruptive behaviours

The results from Model 1 indicate significant differences between SMI groups in relation to disruptive behaviours (HONoS) (see Table 2). As shown in the final step (controlling for age, martial status, and homelessness), women inpatients with a psychotic disorder were 2 times (*p* < 0.001), whilst those with manic disorder were 4.5 times (*p* < 0.001), more likely to have been reported to have exhibited disruptive behaviours during their admission, compared to inpatients with major mood disorders. These values did not differ significantly from step 1, demonstrating that the relationship between SMI groups and disruptive behaviour is robust. With regard to the covariates, those who engaged in substance misuse were 2.2 times more likely to be disruptive (*p* < 0.001), compared to those who did not. Victimisation also showed a significant, but weak difference: those who were victimised were 1.6 times more likely to be violent (*p* < 0.001), compared to those who were not victimised. The overall model is significant at a 0.001 level according to the Model Chi-square statistic [*X* ^2^ (1, 11) = 281.80, *p* < 0.001]. In addition, the Hosmer and Lemeshow Test was non-significant [*X* ^2^ (1, 8) = 8.28, *p* = 0.41], demonstrating an overall good model fit.

**Table 2:** Models 1, 2, & 3: Adjusted odds ratio of disruptive, violent and deliberate self-harm behaviours for SMI diagnostic groups, control variables, substance misuse, personality disorder, ethnicity, and victimisation

	OR (95 % CI)	OR (95 % CI)	OR (95 % CI)
	Disruptive behaviour	Violent behaviour	Deliberate Self-Harm
1. Severe mental illness^a^			
Psychotic disorder	2.06 (1.74–2.43)***	2.08 (1.58–2.72)***	0.21 (0.18–0.025)***
Manic disorder	4.49 (3.54–5.69)***	4.22 (2.99–5.96)***	0.13 (0.09–0.21)***
2. Control variables			
Age	0.99 (0.99–1.00)	0.89 (0.78–1.01)	0.98 (0.97–0.98)***
Martial status	1.00 (0.92–1.09)	1.68 (1.05–2.71)*	1.10 (0.99–1.23)
Homelessness	0.85 (0.56–1.29)	1.68 (1.05–2.71)*	0.58 (0.26–1.27)
3. Substance misuse	2.20 (1.83–2.66)***	1.31 (0.99–1.73)	1.87 (1.49–2.36)***
4. Personality disorder			
Antisocial PD	1.47 (0.72–3.00)	3.53 (1.70–7.35)***	n/a
Borderline PD	n/a	n/a	1.97 (1.60–2.44)***
5. Ethnicity^b^			
Black	1.17 (1.01–1.35)*	1.34 (1.08–1.65)**	0.48 (0.39–0.60)***
Asian	0.87 (0.65–1.17)	0.57 (0.33–0.97)*	0.63 (0.42–0.96)*
Biracial	1.03 (0.80–1.33)	1.25 (0.87–1.80)	0.87 (0.64–1.20)
6. Victimisation	1.62 (1.32–1.99)***	2.40 (1.86–3.09)***	0.83 (0.60–1.15)

*OR* odds ratio, *n/a* not applicable

^a^Reference group major mood disorders

^b^Reference group white

* *P* < 0.05, ** <0.01, *** <0.001

### Violent behaviours

The results for Model 2 include the same IVs as Model 1 (see Table 2). As shown in the final step (controlling for age, martial status, and homelessness), women inpatients with a psychotic disorder were 2 times (*p* < 0.001), whereas those with a manic disorder were 4.2 times, more likely to be violent (*p* < 0.001), compared to those inpatients with major mood disorder. These values did not differ greatly from step 1, again showing that the relationship between SMI groups and violent behaviour is robust. In contrast to the previous model, substance misuse was not significant in the final step of Model 2. Furthermore, ASPD was significant, indicating that those with ASPD were 3.5 times more likely to be violent (*p* < 0.001), compared to those without ASPD. As with the previous model, there was a significant, but weak difference between ethnic groups. Those of Black ethnicity were 1.3 times more likely to be violent (*p* = 0.008), compared to those of White ethnicity. Victimisation also showed a significant difference: those who were victimised were 2.4 times more likely to be violent (*p* < 0.001), compared to those who were not victimised. The overall model is significant at a 0.001 level according to the Model Chi-square statistic [*X* ^2^ (1, 11) = 197.27, *p* < 0.001]. In addition, the Hosmer and Lemeshow Test was non-significant [*X* ^2^ (1, 8) = 12.27, *p* = 0.140], demonstrating an overall good model fit.

### Deliberate self-harm

The results for Model 3 include all the IVs in Models 1 and 2, except BPD was substituted for ASPD, given that the outcome variable for this model is deliberate self-harm (see Table 2). As shown in the final step (controlling for age, martial status, and homelessness), the odds of those with major mood disorder engaging in deliberate self-harm is 7.5 times greater compared to those with psychotic disorders (*p* < 0.001) and 4.8 times greater (*p* < 0.001) compared to those with manic disorders.

With regard to the significant covariates, those who engaged in substance misuse were 1.9 times more likely to reportedly engage in deliberate self-harm (*p* < 0.001), compared to those who did abuse drugs. Those with BPD were two times more likely to engage in self-harm (*p* < 0.001) compared to those without BPD. Furthermore, those of White ethnicity were 2.1 (*p* < 0.001) and 1.6 (*p* < 0.05) times more likely to engage in deliberate self-harm compared to those of Black and Asian ethnicity, respectively. The overall model is significant at a 0.001 level according to the Model Chi-square statistic [*X* ^2^ (1, 11) = 746.30, *p* < 0.001). In addition, the Hosmer and Lemeshow Test was non-significant [*X* ^2^ (1, 8) = 4.19, *p* = 0.839], demonstrating an overall good model fit.

## Discussion

Within the current study, likelihood percentages for disruptive behaviours were higher compared to Grassi et al.’s [40] study (43.3 and 26.5 %) for manic and psychotic disorders, respectively. However, the rates of reported violent behaviours were lower (10.3 and 17.7 %) for manic and psychotic disorders, respectively, although still supported our main hypothesis in this study. This disparity may be attributed to the seriousness of behaviours measured by both variables (e.g., the violent acts variable likely captured more extreme acts of physical violence). Whilst the relationship between psychotic disorders and violence can be explained based on the previous literature and theory, the relationship between manic disorders and violence is not as thoroughly researched and not directly related to a particular theory. Based on the characteristics of manic symptomatology (e.g., grandiose beliefs, racing thoughts, impaired judgment, psychomotor agitation, and impulsiveness), it could be argued that these features mirror in part those of many psychotic disorders.

With regard to the covariates in this study, substance misuse did not have a mediating effect on the relationship between SMI on disruptive and violent behaviours, as the odds ratio from step 1 to step 6 did not significantly increase with the inclusion of substance misuse. Instead, substance misuse showed to be a dynamic (or moderating) risk factor, congruent with earlier findings [2, 41]. The location of the current study offers a possible explanation why this finding differs from the previous large-scale community studies [4]. Moreover, it is also possible that those women in inpatient facilities have more severe SMI symptoms, and therefore, the relationship between SMI and physical violence does not depend on and/or is overly influenced by particular covariates. Personality disorder is also an important variable when assessing risk of violence [22]. In particular, ASPD is important to assess, given its relationship to SMI and also to physical violence [42, 43]. Surprisingly, the prevalence of ASPD was extremely low within this sample, with barely 1 % of the overall sample having documented ASPD. This may be the characteristic of this particular sample and/or a lack of awareness and/or assessment of Axis-II comorbidity when documenting PD in patient’s electronic files. Regardless, results demonstrated a significant independent effect: those with ASPD were 3.5 times more likely to be violent to others compared to those without ASPD (whilst controlling for all other variables). This finding was present only for reported violent behaviours but not disruptive behaviours. Similar to the previous findings [43], the current study also found that individuals with Black ethnic backgrounds significantly predicted disruptive behaviours and physical violence (compared to those of White ethnic backgrounds). Earlier studies [1, 10] also showed an independent increased risk of violence within ethnic minority groups (i.e., African-Caribbean), congruent with the findings of the current study. This observation can be interpreted in many ways, such as staff biases/stereotyping of individuals of different ethnic backgrounds, true higher rates of disruptive and violent behaviours, elevated levels of fear and mistrust held by black ethnic minority groups, and socioeconomic factors that also conflate with both minority populations and risk of violence. Finally, victimisation was shown to have an independent effect, congruent with the previous research findings [26–29] on risk of disruptive and violent behaviours. Moreover, it is important to note that of the total sample of women they were as likely to be harmed by others (victimised; 9.3 %), as they were to harm others (violent behaviours; 9.5 %). These findings reiterate the importance of including victimisation when evaluating the likelihood of violence, as the relationship between the early or current victimisation and later violence can help researchers better understand later violence from a developmental perspective. Taken together, our results showed to also partly support our second hypothesis—substance abuse and ethnicity did in fact moderate the main relationship, whilst the ASPD and victimisation had an independent effect.

The current findings of this study also complement the previous literature that demonstrates a strong relationship between major mood disorders and deliberate self-harm, in comparison with psychotic and manic disorders. Furthermore, it is notable that whilst the overall women were more likely to harm themselves (13.5 %) than others (9.2 %), this was not the case for those with psychotic and particularly manic disorders who were at elevated risk of harming others than themselves, which supported our third hypothesis. This study also found that substance misuse was significantly related to self-harm, in addition to BPD, congruent with Haw et al.’s previous studies [44, 45]. In addition to these variables, ethnic backgrounds were also significant, with those women of White ethnic backgrounds being significantly more likely to engage in self-harm, as compared to those of Black and Asian ethnic backgrounds. This variable was added into the model for exploratory purposes and subsequently highlights the differences between ethnic groups in relation to physical violence to others and oneself (self-harm). This difference is important to consider, given that there may be a hidden covariate partially responsible for this difference (e.g., cultural differences or racial biases between patients and staff, etc.). The current findings compliment those of a recent epidemiological study [46] which also found that women were more likely to harm themselves than harm others (particularly if they had a major mood disorder) and that those individuals of Black ethnicity were less likely to engage in deliberate self-harm than their White counterparts.

Limitations of the present study include issues surrounding missing data, in particular with the PD variables. This information was randomly missing, as health officials who entered the data in CRIS, either forgot to enter in new information or forgot to ask the patients to fill out the questionnaires. Methods, such as mean imputation and regression imputation, were not used as they fail to account for the variability that is present in the hypothetical data values [47]. In effect, particular cases were excluded (via a data filter) and, therefore, may have caused a bias in the sample. The frequency of physical violence per case was also not documented (i.e., if a case was documented as violent, they may have only had one act of physical violence or numerous). Specific significant variables in the previous literature were not able to be included in the current study due to this information not be available through CRIS (e.g., specific psychotic features, delusional distress, and medication use). Future research should address these limitations and also assess these relationships within men inpatients and compare gender differences. As women are less emphasised in many research domains, men are understudied when addressing self-harm. This study was also characterised by a number of strengths, such as validated measures for key variables, design (consideration of temporal proximity), and use of a large-scale database.

## Conclusions

The current study supported earlier findings [14, 15, 46] and the majority of hypotheses tested. Women with psychotic, and particularly manic disorders, were at increased odds of engaging in disruptive and violent behaviours compared to those with major mood disorders. Substance misuse, Black ethnicity, ASPD, and victimisation conferred significant independent risk factors for disruptive and violent behaviours. Conversely, women with major mood disorders were at increased odds of deliberate self-harm compared to those with psychotic and manic disorders with substance misuse, BPD, and White ethnicity conferring independent risk factors. The current findings address the need to further analyse explanatory relationships between diagnostic subgroups and violent typologies among women with severe mental illness.
